# Supplementary material for: The determinants of genetic diversity in butterflies
Source: Nat Commun. 2019 Aug 1;10:3466. doi: 10.1038/s41467-019-11308-4 (PMC6672018; doi:10.1038/s41467-019-11308-4)
Supplement: Supplementary file 1 — Supplementary Information [file 41467_2019_11308_MOESM1_ESM.pdf]

Supplementary Information

The determinants of genetic diversity in butterflies

Mackintosh et al.

Contents

Supplementary Table 1 3

Supplementary Figures 1–9 4

Supplementary Methods 13

Data QC and *de novo* transcriptome assembly 13

Variant calling 13

Estimating genetic diversity 14

Estimation of π for mitochondrial COI locus 14

Phylogeny reconstruction 15

Supplementary Note 1 16

Supplementary References 18

Supplementary Table 1 Correlates of genetic diversity inferred under a maximal model

Table 1:

Predictor	Response	Posterior mean slope*	95% CI	p_{MCMC}**
Census population size	π_{4D}	0.058	-0.153, 0.274	0.581
Census population size	π_{0D}	0.044	-0.092, 0.175	0.507
LHP breadth (poly.)	π_{4D}	-0.291	-0.787, 0.188	0.215
LHP breadth (poly.)	π_{0D}	-0.120	-0.443, 0.189	0.433
Body size	π_{4D}	-0.274	-0.530, -0.036	0.036
Body size	π_{0D}	-0.184	-0.340, -0.009	0.031
Relative egg size	π_{4D}	-0.078	-0.394, 0.212	0.595
Relative egg size	π_{0D}	0.020	-0.154, 0.223	0.833
Voltinism (poly.)	π_{4D}	0.324	-0.162, 0.757	0.159
Voltinism (poly.)	π_{0D}	0.139	-0.151, 0.444	0.349
Chrom. number	π_{4D}	0.258	0.058, 0.463	0.016
Chrom. number	π_{0D}	0.131	-0.000, 0.263	0.056
Genome size	π_{4D}	0.053	-0.186, 0.270	0.648
Genome size	π_{0D}	0.078	-0.072, 0.235	0.295

* posterior mean estimates of the slope of linear correlates of genetic diversity at synonymous (π_{4D}) and non-synonymous (π_{0D}) sites under a maximal model. For discrete predictors (LHP breadth and voltinism) the level of the factor described in the table is indicated in brackets

** twice the probability that the posterior mean slope estimate is > 0 or < 0

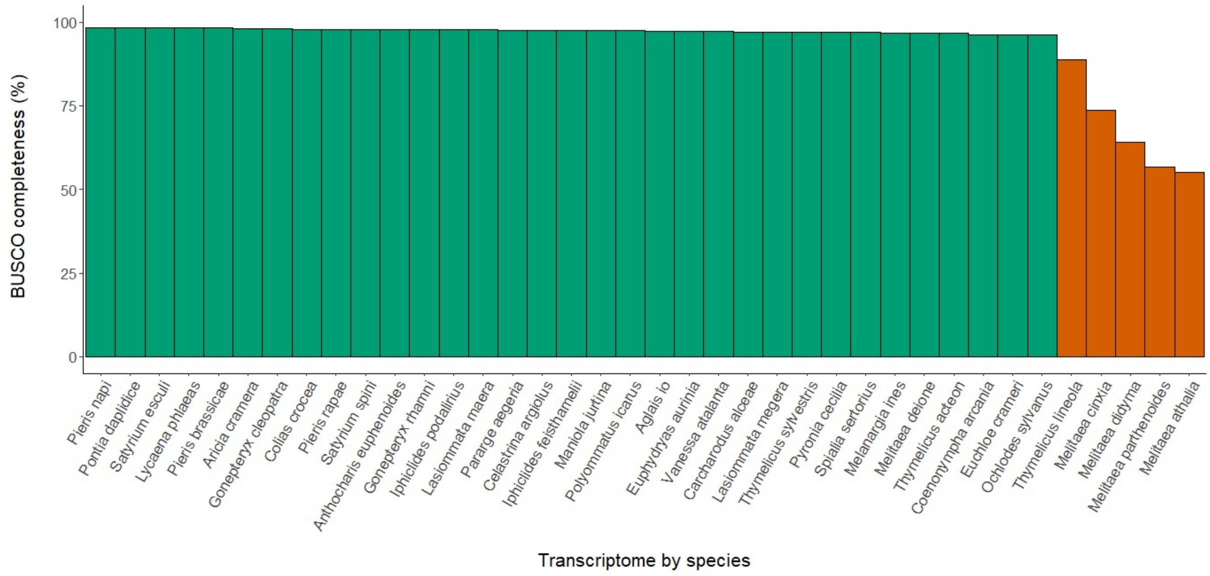

Supplementary Figure 1 Completeness of transcriptome assemblies assessed using BUSCO scores. Transcriptomes assembled *de novo* as part of this study are shown in green, assemblies based on data from Romiguier et al. (2014) are shown in orange.

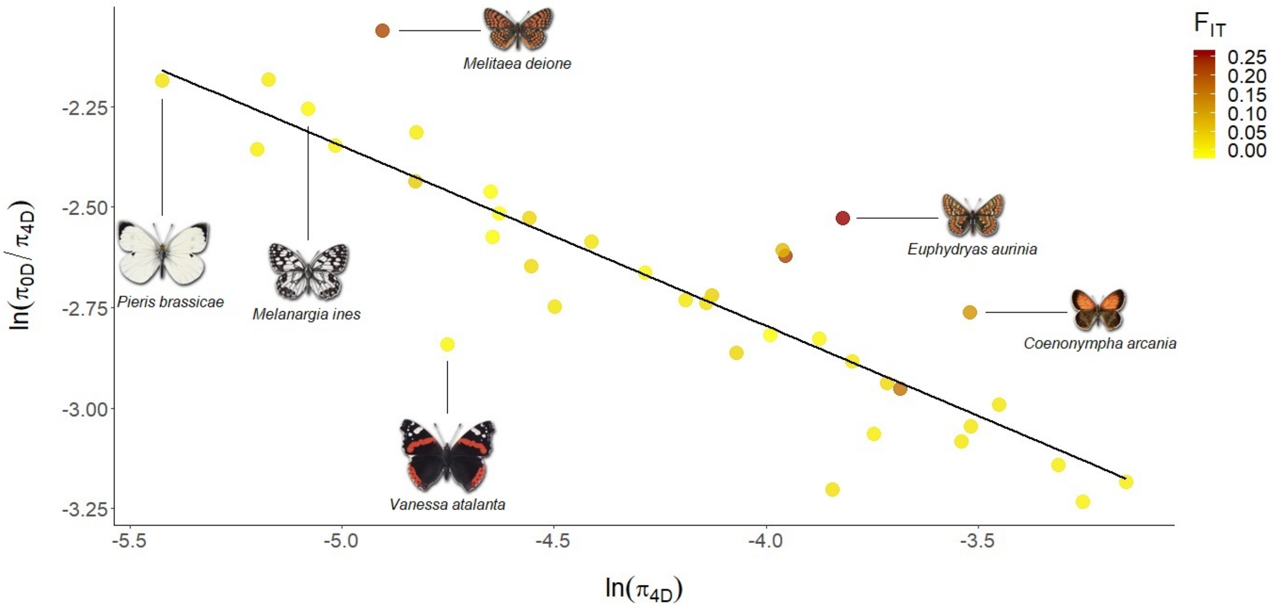

Supplementary Figure 2 The negative relationship between genetic diversity, $ln(\pi_{4D})$ and selection efficacy $ln(\pi_{0D}/\pi_{4D})$. A number of species with high genetic differentiation (F_{IT}) fall above the line of best fit, i.e. they have less efficient selection than expected. In contrast, the migratory species *Vanessa atalanta* falls below the line of best fit. The two species with the lowest chromosome numbers (*Pieris brassicae* and *Melanargia ines*) both fall close to the line of best fit, suggesting that they do not have a lower mutation rate than other butterfly species.

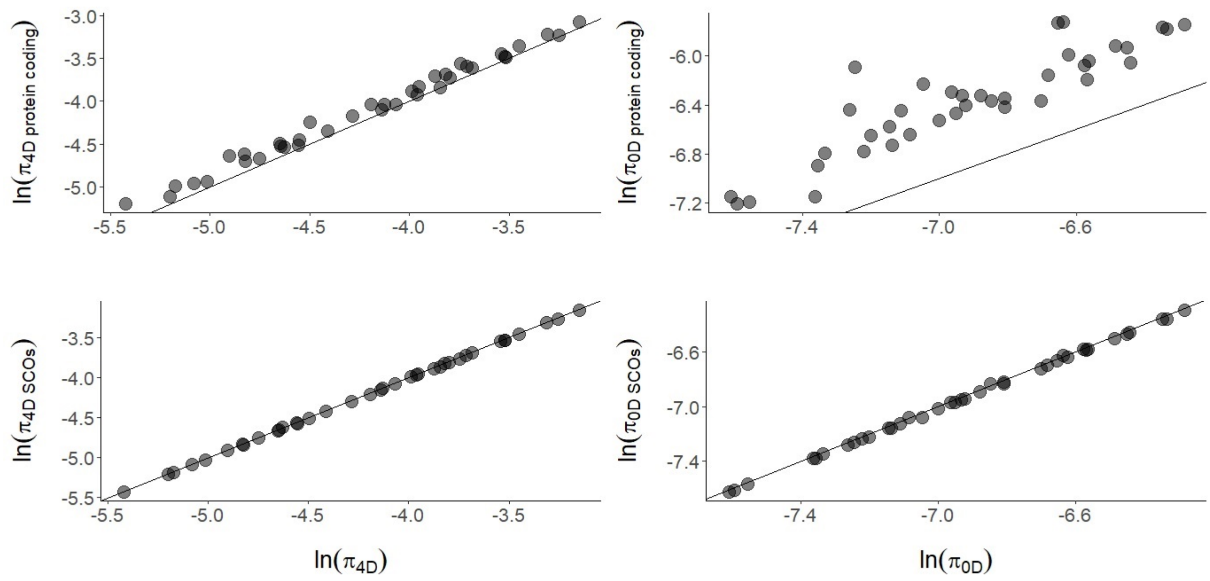

Supplementary Figure 3 Estimates of genetic diversity at synonymous sites (π_{4D}, left) and non-synonymous sites (π_{0D}, right) are higher when based on all protein coding transcripts, i.e. without removing non-orthologous and putative Z-linked transcripts (top). Removing putative Z-linked transcripts from the set of orthologous transcripts has a negligible effect on estimates of π_{4D} and π_{0D} (bottom). The effect of both filters is much smaller for π_{4D} than π_{0D}.

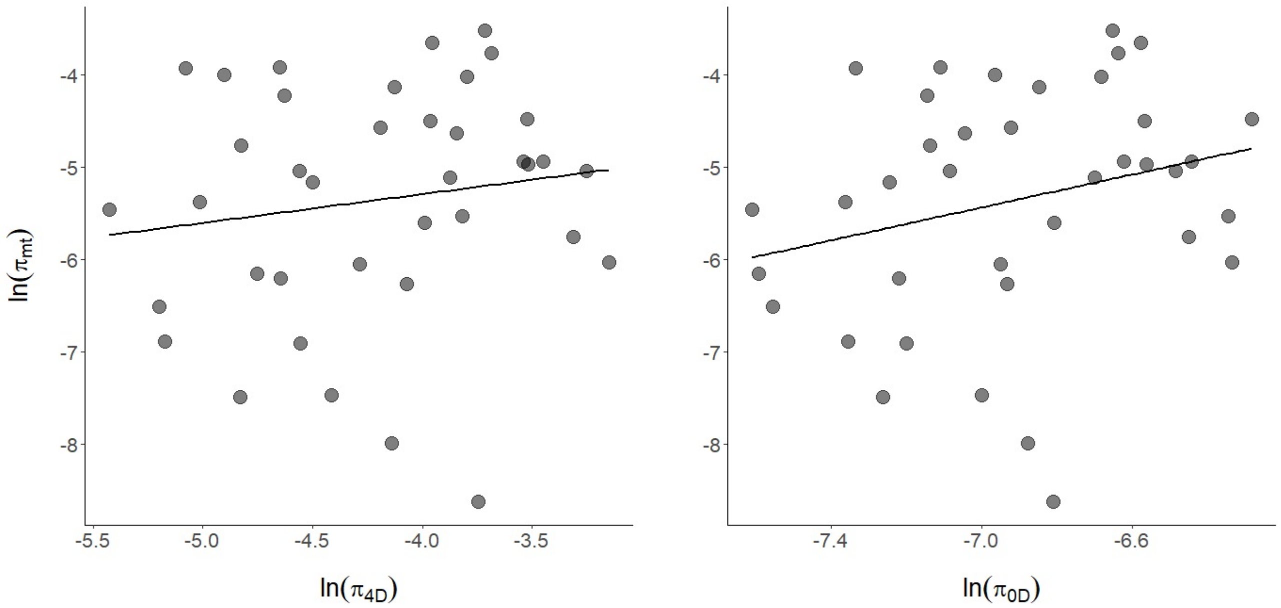

Supplementary Figure 4 Mitochondrial diversity at the CO1 locus is not significantly correlated with nuclear diversity both at synonymous (π_{4D}, left) and non-synonymous (π_{0D}, right) sites.

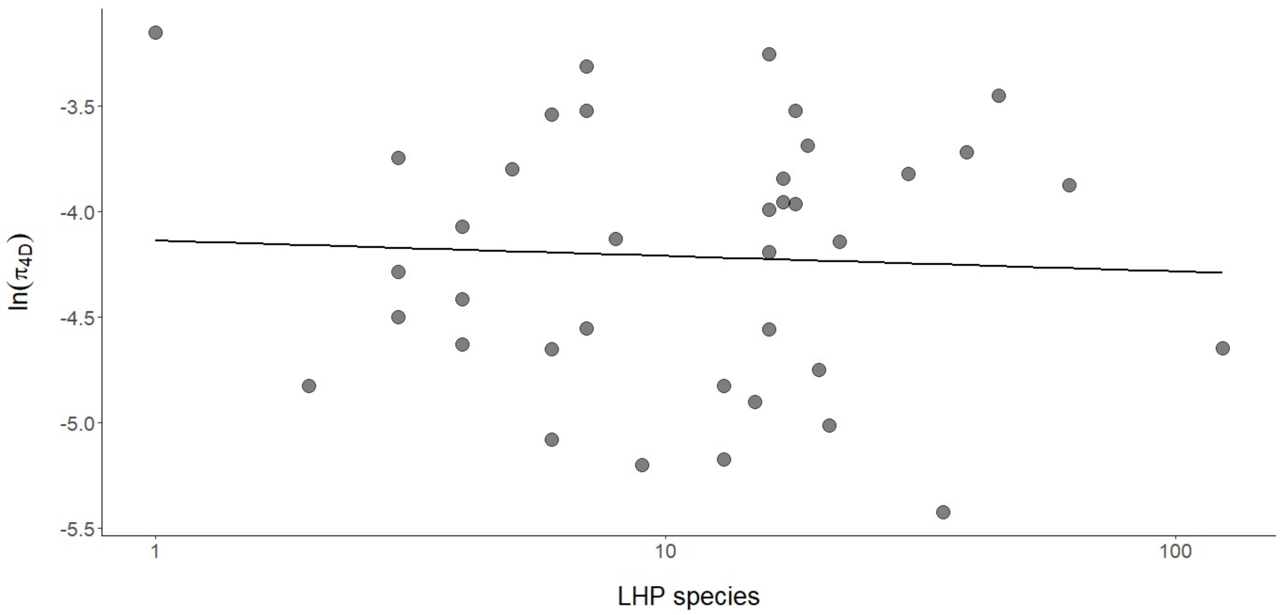

Supplementary Figure 5 There is no significant correlation between the number of larval host plant (LHP) species a butterfly species uses and its genome-wide neutral genetic diversity.

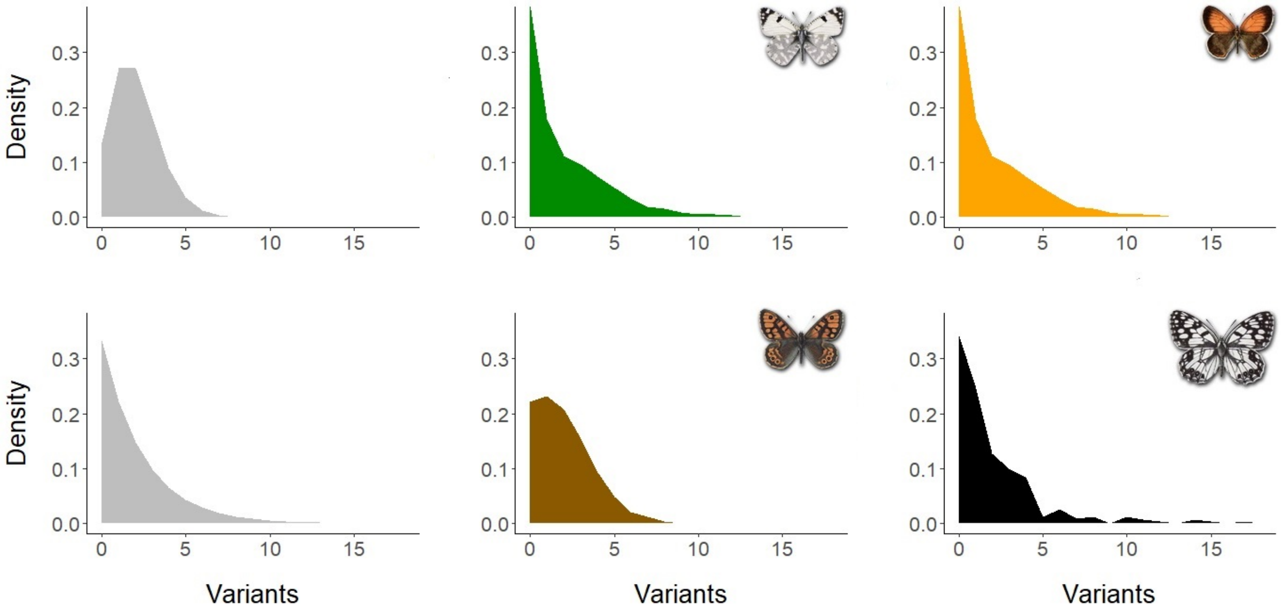

Supplementary Figure 6 The distribution of heterozygous sites (S) in sequence blocks of a fixed length (4D sites only) contains information about past demography: the expected distribution under extreme population growth (top left) (starshaped genealogy) or a model of constant N_e (bottom left). *Coenonympha arcania* (top right) fits the distribution expected under a model of constant N_e best, *Lasiommata megera* (bottom middle) is closest to the distribution expected a population with a history of rapid growth. In most species $Var[S]$ is closer to the prediction for a constant N_e rather than extreme growth, e.g. *Euchloe crameri* (top left) represents the median (in terms of $Var[S]$) in the dataset. *Melanargia ines* (bottom right) is the species with the highest $Var[S]$.

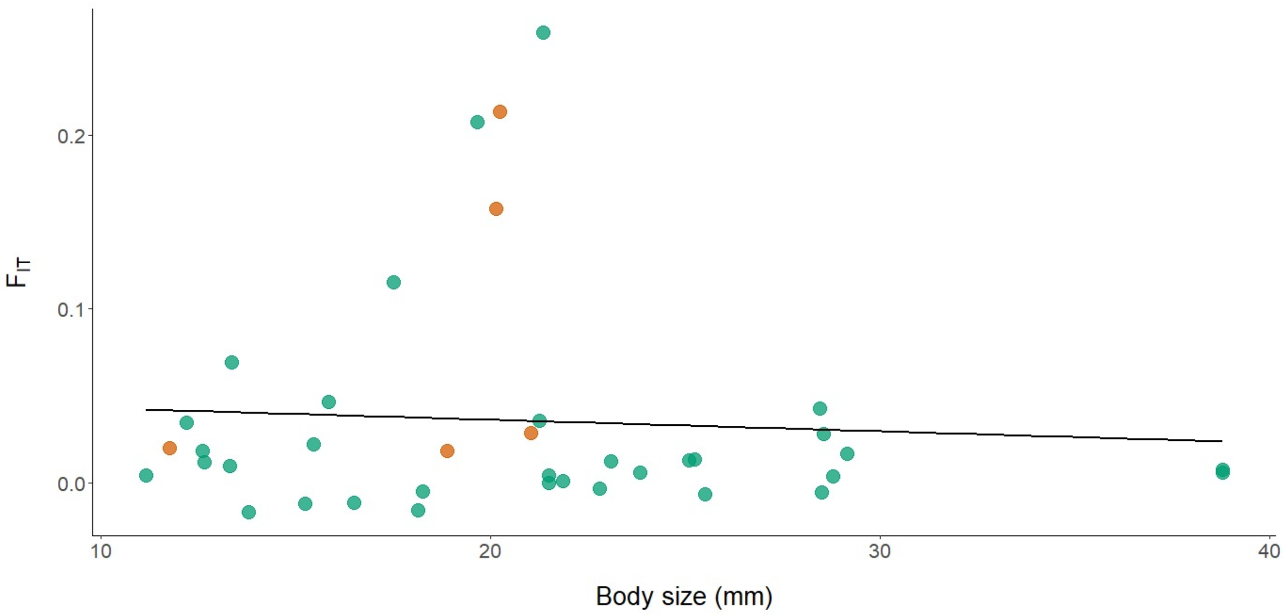

Supplementary Figure 7 Genetic differentiation between individuals (F_{IT}) sampled from different regions of Iberia is not correlated with body size. Species sampled within Iberia are shown in green, those sampled by Romiguier et al. (2014) outside of Iberia are shown in orange.

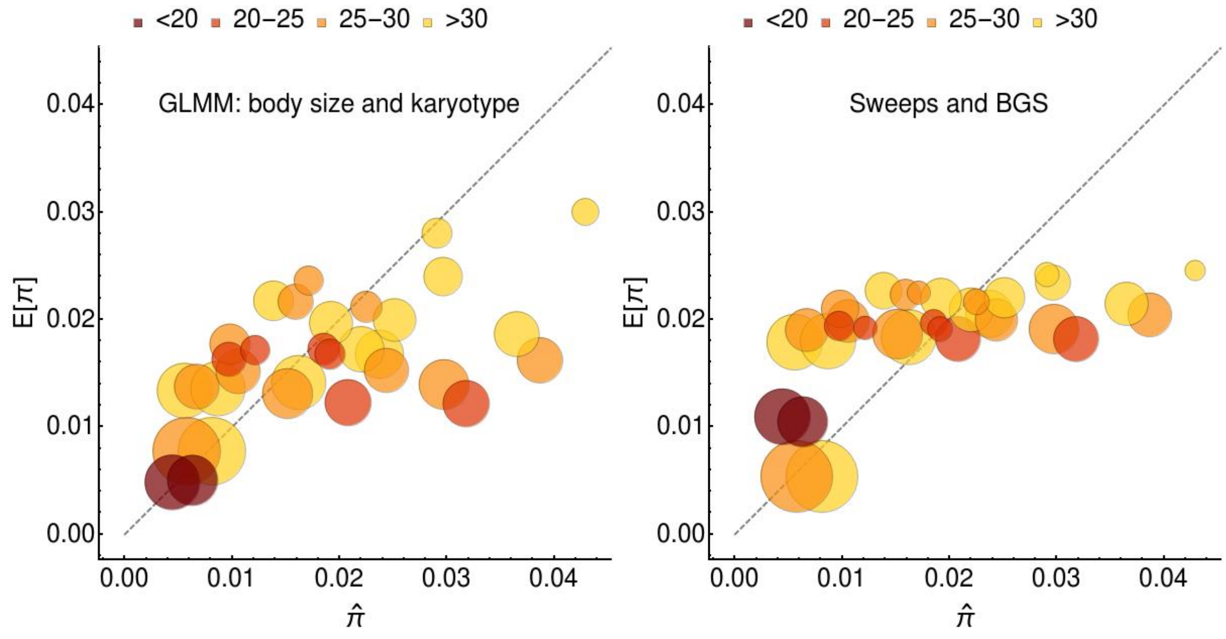

Supplementary Figure 8 The minimal model inferred using MCMCglmm predicts the observed π_{4D} ($\hat{\pi}$) as well as an explicit model of the effect of selection on linked neutral diversity. Circles are proportional to the body size of each species, the colour indicates chromosome number.

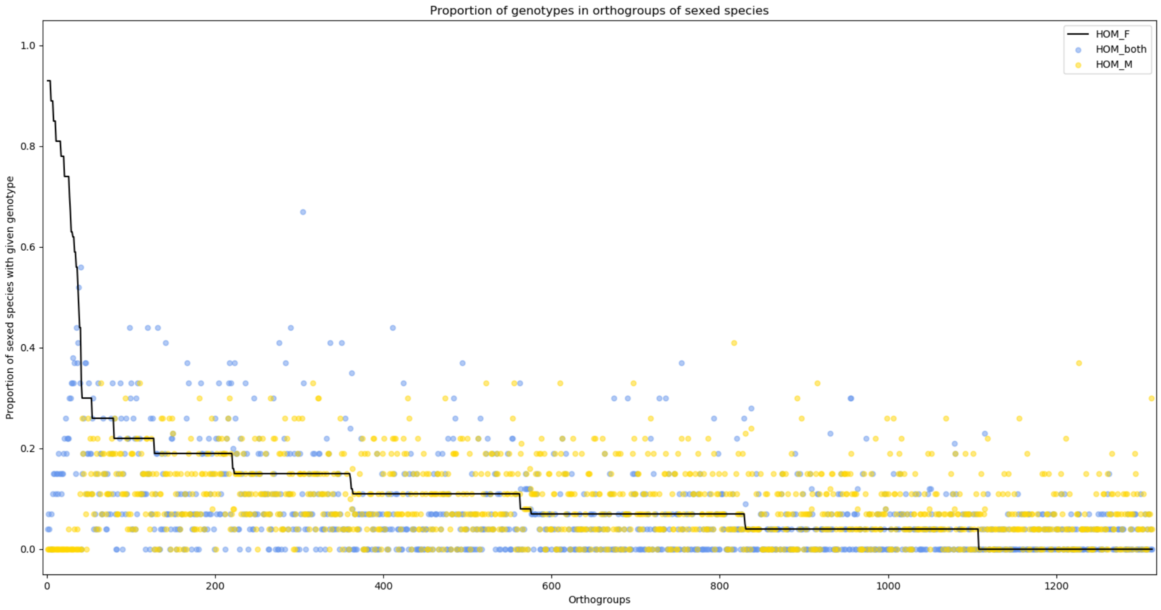

Supplementary Figure 9 The proportion of species (out of a total of 27 species in which one male and one female were sampled, see Supplementary Data 1) in which female samples are homozygous (HOM_F); male samples are homozygous (HOM_M) or both sexes are homozygous (HOM_{both}) for each single copy orthologue cluster.

Supplementary Methods

Data QC and *de novo* transcriptome assembly

Raw read quality was evaluated using `FastQC v0.11.7` (Andrews, 2015) and visualised with `MultiQC v1.5a` (Ewels et al., 2016). Illumina adapters were removed, reads trimmed using `Trimmomatic` (Bolger et al., 2014) (using default parameters) and only reads of length ≥25b were retained. Transcriptomes were assembled *de novo* from both individuals of each species with `Trinity` (Haas et al., 2013). Assembly completeness was assessed using `BUSCO v3.0.2` (Simão et al., 2015) together with the Insecta database `insectodb9` (1,658 single copy orthologues from 42 insect species) as a reference (Supplementary Figure 1).

Variant calling

Protein coding transcripts were identified using `Transdecoder` (Haas and Papanicolaou), `BLAST` (Altschul et al., 1990) and `HMMER` (Eddy and the HMMER development team, 2018). `Transdecoder` was used to find open reading frames (ORFs) within transcripts, while homology searches were done using `BLAST` and `HMMER` to identify transcripts containing known sequences and domains. Finally, the predict function in `Transdecoder` was used to score likely protein coding transcripts based on ORF presence and homology. For each species, reads of both individuals were separately mapped to the CDS of the longest isoform of complete proteins using `BWA MEM` (Li, 2013). Loci which were suitable for variant calling were selected using the callable loci function in `GATK` (McKenna et al., 2010). We selected sites with a read depth ≥ 10 and MQ ≥ 1. Callable loci were intersected between individuals using `BEDTools` (Quinlan and Hall, 2010), removing sites that were not expressed in both individuals sampled in each species. Variants were called using `Freebayes` (Garrison and Marth, 2012), and only retained if supported by more than three reads,

with the variant being found in both the $3^{'}$ and $5^{'}$ end of reads, as well as in both forward and reverse reads. Excluded variants were masked for downstream analysis, and so did not contribute to the total length.

Estimating genetic diversity

Protein clustering using `Orthofinder` (Emms and Kelly, 2015) revealed 1,314 single copy orthologue (SCO) clusters in the 33 transcriptomes with high completeness (`BUSCO` scores 96.3 – 98.4%, Supplementary Figure 1). Only transcripts corresponding to SCOs were used to estimate π in each species. The inclusion of Z-linked genes in this estimation is potentially problematic. To identify and filter out putative Z-linked SCOs, we made use of the fact that these would be expected to be homozygous in all female individuals. For each SCO we tallied the number of species (considering only the 27 species for which both sexes were sampled, Supplementary Data 1) in which only the female sample is homozygous (HOM_F), only the male sample is homozygous (HOM_M) or both samples are homozygous (HOM_{both}) (across all sites) (Supplementary Figure 9). None of the 1,314 conserved SCO clusters contains transcripts that are HOM_F in all 27 species. However, since we do not know how conserved Z-linkage of genes is across these species, this filter is not sufficient. We therefore removed 37 SCO clusters (2.8 % of the data) for which $\geq$ 50% species were HOM_F. This has almost no effect on estimates of π (Supplementary Figure 3).

Estimation of π for mitochondrial COI locus

Mitochondrial π was calculated for the COI barcode locus using sequences retrieved from the BOLD systems database (Ratnasingham and Hebert, 2007). Alignments of 658bp for each species were produced in `Bioedit` (Hall, 1999) using CLUSTAL-W (Thompson et al., 1994) and manual inspection. Mean pairwise π of each alignment was calculated in `MEGA7` (Stecher et al., 2016).

Phylogeny reconstruction

Single-copy orthologous proteins present in all transcriptome assemblies (including the five species sequenced by Romiguier et al. (2014)) — as well as the genome of the silkmoth *Bombyx mori* — were identified with `Orthofinder`. The resulting 218 protein sequences were concatenated for each species, aligned using `MAFFT` (Katoh and Standley, 2013), and trimmed using `trimAl` (Capella-Gutiérrez et al., 2009). The final alignment contained 59,747 amino acid sites, 22,429 of which were informative for phylogenetic inference. 20 maximum likelihood (ML) tree searches were conducted using the substitution model PROTGTR+GAMMA with `RAxML` (Stamatakis, 2014). To assess confidence in the ML tree, non-parametric bootstrap values were obtained from 100 replicates.

Supplementary Note 1

The effect of selection on linked sites

We use the equation derived by Barton (2000, eq. 1), modified for the case of semi-dominant favourable mutation with selection coefficient s when homozygous. This gives the reduction in neutral diversity immediately following a sweep, measured relative to its initial value, as $\Delta\pi \approx (2N_e s)^{-(4r/s)}$, where r is the frequency of recombination between the neutral and selected sites (for alternative derivations, see Coop and Ralph (2012); Weissman and Barton (2012); Elyashiv et al. (2016)). This can be written as $\Delta\pi \approx (2N_e s)^{-(2r/J)}$, where $J = s/[2\ln(2N_e s)]$. Following Weissman and Barton (2012), assume that r is given by a linear function of physical distance z between sites, $r = r_c z$. This is reasonable if recombination is due solely to crossing over, and sweep effects extend over relative small distances, so that double crossing over can be neglected. If, however, gene conversion also contributes to recombination, as is likely, the effect of a sweep is considerably reduced compared with this expression (Campos et al., 2017).

Following Kaplan et al. (1989) and Wiehe and Stephan (1993), $\Delta\pi$ can be equated to the probability that a sweep results in a coalescent event. Such an event is assumed to be instantaneous compared with coalescent events caused by genetic drift, whose rate is $1/(2N_0)$ in the absence of selective effects. If adaptive substitutions occur at a rate ν per basepair per generation, and there is no Hill-Robertson interference among them, the rate of sweep-induced coalescent events at a focal neutral site caused by all sweeps to the right and left of this site can be derived by treating the chromosome as a continuum, and integrating over all contributing sites. Following Weissman and Barton (2012), this approach gives the net rate of sweep-induced coalescent events per unit per unit coalescent time ($2N_0$ generations) as

$$C_s = 2N_0\nu \int_{-\infty}^{\infty} \exp(-2r_c z/J)\mathrm{d}z = 4N_0\nu \int_{0}^{\infty} \exp(-2r_c z/J)\mathrm{d}z = 2N_0\nu J r_c^{-1} \quad (1)$$

If we assume that there are n_c chromosomes, each with a map length of 0.25 Morgans after taking the lack of crossing over in males into account, and a total haploid genome size of G basepairs, we have $r_c = n_c/(4G)$. The total rate of substitutions per genome is $\nu_T = G\nu$. We thus have:

$$C_s \approx 8N_0 G\nu J n_c^{-1} = 8N_0 \nu_T J n_c^{-1} \tag{2}$$

Supplementary References

Altschul, S. F., Gish, W., Miller, W., Myers, E. W., and Lipman, D. J. (1990). Basic local alignment search tool. *Journal of Molecular Biology*, 215(3):403 – 410.

Andrews, S. (2015). FastQC a quality-control tool for high-throughput sequence data.

Barton, N. H. (2000). Genetic hitchhiking. *Philosophical Transactions of the Royal Society of London B: Biological Sciences*, 355(1403):1553–1562.

Bolger, A. M., Lohse, M., and Usadel, B. (2014). Trimmomatic: a flexible trimmer for illumina sequence data. *Bioinformatics*, 30(15):2114–2120.

Campos, J. L., Zhao, L., and Charlesworth, B. (2017). Estimating the parameters of background selection and selective sweeps in Drosophila in the presence of gene conversion. *Proceedings of the National Academy of Sciences*, 114(24):E4762–E4771.

Capella-Gutiérrez, S., Silla-Martínez, J. M., and Gabaldón, T. (2009). trimAl: a tool for automated alignment trimming in large-scale phylogenetic analyses. *Bioinformatics (Oxford, England)*, 25(15):1972–1973.

Coop, G. and Ralph, P. (2012). Patterns of neutral diversity under general models of selective sweeps. *Genetics*, 192(1):205–224.

Eddy, S. R. and the HMMER development team (2018). HMMER: biosequence analysis using profile hidden Markov models.

Elyashiv, E., Sattath, S., Hu, T. T., Strutsovsky, A., McVicker, G., Andolfatto, P., Coop, G., and Sella, G. (2016). A genomic map of the effects of linked selection in Drosophila. *PLOS Genetics*, 12(8):1–24.

Emms, D. M. and Kelly, S. (2015). OrthoFinder: solving fundamental biases in whole genome comparisons dramatically improves orthogroup inference accuracy. *Genome Biology*, 16(1):157.

Ewels, P., Magnusson, M., Lundin, S., and Käller, M. (2016). MultiQC: summarize analysis results for multiple tools and samples in a single report. *Bioinformatics*, 32(19):3047–3048.

Garrison, E. and Marth, G. (2012). Haplotype-based variant detection from short-read sequencing. *ArXiv e-prints*.

Haas, B. and Papanicolaou, A. Transdecoder (find coding regions within transcripts).

Haas, B. J., Papanicolaou, A., Yassour, M., Grabherr, M., Blood, P. D., Bowden, J., Couger, M. B., Eccles, D., Li, B., Lieber, M., MacManes, M. D., Ott, M., Orvis, J., Pochet, N., Strozzi, F., Weeks, N., Westerman, R., William, T., Dewey, C. N., Henschel, R., LeDuc, R. D., Friedman, N., and Regev, A. (2013). De novo transcript sequence reconstruction from RNA-seq using the Trinity platform for reference generation and analysis. *Nature Protocols*, 8:1494.

Hall, T. A. (1999). BioEdit: a user-friendly biological sequence alignment editor and analysis program for Windows 95/98/NT. *Nucleic Acids Symposium Series*, 41:95–98.

Kaplan, N. L., Hudson, R. R., and Langley, C. H. (1989). The "hitchhiking effect" revisited. *Genetics*, 123(4):887–899.

Katoh, K. and Standley, D. M. (2013). MAFFT multiple sequence alignment software version 7: Improvements in performance and usability. *Molecular Biology and Evolution*, 30(4):772–780.

Li, H. (2013). Aligning sequence reads, clone sequences and assembly contigs with BWA-MEM.

McKenna, A., Hanna, M., Banks, E., Sivachenko, A., Cibulskis, K., Kernytsky, A., Garimella, K., Altshuler, D., Gabriel, S., Daly, M., and DePristo, M. A. (2010). The Genome Analysis Toolkit: A MapReduce framework for analyzing next-generation DNA sequencing data. *Genome Research*, 20(9):1297–1303.

Quinlan, A. R. and Hall, I. M. (2010). BEDTools: a flexible suite of utilities for comparing genomic features. *Bioinformatics*, 26(6):841–842.

Ratnasingham, S. and Hebert, P. D. N. (2007). bold: The Barcode of Life Data System (http://www.barcodinglife.org). *Mol Ecol Notes*, 7(3):355–364.

Romiguier, J., Gayral, P., Ballenghien, M., Bernard, A., Cahais, V., Chenuil, A., Chiari, Y., Dernat, R., Duret, L., Faivre, N., Loire, E., Lourenco, J. M., Nabholz, B., Rou, C., Tsagkogeorga, G., Weber, A. A.-T., Weinert, L. A., Belkhir, K., Bierne, N., Glémin, S., and Galtier, N. (2014). Comparative population genomics in animals uncovers the determinants of genetic diversity. *Nature*, 515:261–263.

Simão, F. A., Waterhouse, R. M., Ioannidis, P., Kriventseva, E. V., and Zdobnov, E. M. (2015). BUSCO: assessing genome assembly and annotation completeness with single-copy orthologs. *Bioinformatics*, 31(19):3210–3212.

Stamatakis, A. (2014). RAxML version 8: a tool for phylogenetic analysis and post-analysis of large phylogenies. *Bioinformatics*, 30(9):1312–1313.

Stecher, G., Kumar, S., and Tamura, K. (2016). MEGA7: Molecular Evolutionary Genetics Analysis Version 7.0 for Bigger Datasets. *Molecular Biology and Evolution*, 33(7):1870–1874.

Thompson, J. D., Higgins, D. G., and Gibson, T. J. (1994). Clustal w: improving the sensitivity of progressive multiple sequence alignment through sequence weighting, position-specific gap penalties and weight matrix choice. *Nucleic Acids Res*, 22(22):4673–4680.

Weissman, D. B. and Barton, N. H. (2012). Limits to the rate of adaptive substitution in sexual populations. *PLOS Genetics*, 8(6):1–18.

Wiehe, T. H. and Stephan, W. (1993). Analysis of a genetic hitchhiking model, and its application to DNA polymorphism data from Drosophila melanogaster. *Molecular Biology and Evolution*, 10(4):842–854.
