# Supplementary material for: The determinants of genetic diversity in butterflies
Source: Nat Commun. 2019 Aug 1;10:3466. doi: 10.1038/s41467-019-11308-4 (PMC6672018; doi:10.1038/s41467-019-11308-4)
Supplement: Supplementary file 3 — Reporting Summary [file 41467_2019_11308_MOESM3_ESM.pdf]

nature research

Corresponding author(s): Konrad Lohse
Alex Mackintosh

Last updated by author(s): May 26, 2019

# Reporting Summary

Nature Research wishes to improve the reproducibility of the work that we publish. This form provides structure for consistency and transparency in reporting. For further information on Nature Research policies, see Authors & Referees and the Editorial Policy Checklist .

## Statistics

For all statistical analyses, confirm that the following items are present in the figure legend, table legend, main text, or Methods section.

| n/a | Confirmed | |
|---|---|---|
| ☐ | ☒ | The exact sample size (*n*) for each experimental group/condition, given as a discrete number and unit of measurement |
| ☐ | ☒ | A statement on whether measurements were taken from distinct samples or whether the same sample was measured repeatedly |
| ☐ | ☒ | The statistical test(s) used AND whether they are one- or two-sided<br>*Only common tests should be described solely by name; describe more complex techniques in the Methods section.* |
| ☐ | ☒ | A description of all covariates tested |
| ☐ | ☒ | A description of any assumptions or corrections, such as tests of normality and adjustment for multiple comparisons |
| ☐ | ☒ | A full description of the statistical parameters including central tendency (e.g. means) or other basic estimates (e.g. regression coefficient) AND variation (e.g. standard deviation) or associated estimates of uncertainty (e.g. confidence intervals) |
| ☐ | ☒ | For null hypothesis testing, the test statistic (e.g. *F*, *t*, *r*) with confidence intervals, effect sizes, degrees of freedom and *P* value noted<br>*Give P values as exact values whenever suitable.* |
| ☐ | ☒ | For Bayesian analysis, information on the choice of priors and Markov chain Monte Carlo settings |
| ☐ | ☒ | For hierarchical and complex designs, identification of the appropriate level for tests and full reporting of outcomes |
| ☐ | ☒ | Estimates of effect sizes (e.g. Cohen's *d*, Pearson's *r*), indicating how they were calculated |

*Our web collection on statistics for biologists contains articles on many of the points above.*

## Software and code

Policy information about availability of computer code

| | |
|---|---|
| Data collection | RNAseq data from wild caught insects, flow cytometry. Lifehistory and geographic range data from public databases. |
| Data analysis | MCMCglmm R package, Mathematica v. 11, R code and custom python code used for the analysis is available on github. The Mathematica notebook is provided as a Supplementary File. |

For manuscripts utilizing custom algorithms or software that are central to the research but not yet described in published literature, software must be made available to editors/reviewers. We strongly encourage code deposition in a community repository (e.g. GitHub). See the Nature Research guidelines for submitting code & software for further information.

## Data

Policy information about availability of data

All manuscripts must include a data availability statement. This statement should provide the following information, where applicable:

- Accession codes, unique identifiers, or web links for publicly available datasets
- A list of figures that have associated raw data
- A description of any restrictions on data availability

The source data used for MCMCglmm analyses in this study underlying Table 1, Fig. 1 and 2 and Supplementary Table and Supplementary Fig. 2-8 are available from Supplementary Data 3. Raw reads are deposited at the European Nucleotide Archive under project PRJEB31360 [https://www.ebi.ac.uk/ena/data/view/PRJEB31360].

# Field-specific reporting

Please select the one below that is the best fit for your research. If you are not sure, read the appropriate sections before making your selection.

# Ecological, evolutionary & environmental sciences study design

All studies must disclose on these points even when the disclosure is negative.

| | |
|---|---|
| Study description | Genetic diversity a synonymous and non-synonymous sites was estimated from single copy orthologues in transcriptome data. We tested for potential predictors of genetic diversity (a total of 7 predictors defined prior to the analysis) using a general linear model (with phylogeny as a random effect). |
| Research sample | The sample of 33 species for which we generated RNAseq data includes all butterfly taxa for which we managed to collect RNA samples from at least two regions of Iberia in one field season (2017). To the best of our knowledge, the five species from Romiguier et al. (2014) are all European butterfly taxa for which equivalent RNASeq data (more than two wild-caught samples from different regions) were available at the time of the analysis. |
| Sampling strategy | Two replicate individuals (sampled from different populations) were used to estimate genetic diversity in each species. Genome size estimates using flow cytometry are based on two replicate samples which were measured individually. |
| Data collection | Estimated of genetic diversity based on RNASeq data and genome size estimates (obtained using flow cytometry) were generated by this study. Data on life-history traits, body size, species ranges and chromosome number were collated from the literature and public databases. |
| Timing and spatial scale | To control for population genetic structure, estimates of genetic diversity were based on two samples from different regions of Iberia. |
| Data exclusions | No data were excluded. |
| Reproducibility | Raw and final sequence data, the source data for and the code for all statistical analyses are available |
| Randomization | n/a |
| Blinding | n/a |

Did the study involve field work? ☒ Yes ☐ No

## Field work, collection and transport

| | |
|---|---|
| Field conditions | n/a |
| Location | Several localities in Spain, Portugal and France, see Supporting Data 1 for details. |
| Access and import/export | Permissions for field sampling were obtained from the Generalitat de Catalunya (SF/639, 2017), the Gobierno de Aragon (INAGA/500201/24/2018/0614 to Karl Wotton) and the Gobierno del Principado de Asturias (014252, 2017). |
| Disturbance | The study involved minimal sampling of butterflies (4 individuals per species) from the field for genetic analysis. |

# Reporting for specific materials, systems and methods

We require information from authors about some types of materials, experimental systems and methods used in many studies. Here, indicate whether each material, system or method listed is relevant to your study. If you are not sure if a list item applies to your research, read the appropriate section before selecting a response.

## Materials & experimental systems

| n/a | Involved in the study | |
|---|---|---|
| ☒ | ☐ | Antibodies |
| ☒ | ☐ | Eukaryotic cell lines |
| ☒ | ☐ | Palaeontology |
| ☐ | ☒ | Animals and other organisms |
| ☒ | ☐ | Human research participants |
| ☒ | ☐ | Clinical data |

## Methods

| n/a | Involved in the study | |
|---|---|---|
| ☒ | ☐ | ChIP-seq |
| ☐ | ☒ | Flow cytometry |
| ☒ | ☐ | MRI-based neuroimaging |

## Animals and other organisms

Policy information about studies involving animals; ARRIVE guidelines recommended for reporting animal research

| | |
|---|---|
| Laboratory animals | n/a |

| | |
|---|---|
| Wild animals | Butterflies were hand-netted in the field and frozen from live (-80) in a dryshipper. Permissions for field sampling were obtained from the Generalitat de Catalunya (SF/639), the Gobierno de Aragon (INAGA/500201/24/2018/0614 to Karl Wotton) and the Gobierno del Principado de Asturias (014252). Species IDs and sex information are provided in Supplementary Data 1. |
| Field-collected samples | The study did not involve laboratory experiments on field-caught animals. |
| Ethics oversight | Field sampling of butterflies was conducted in compliance with the School of Biological Sciences Ethics Committee at the University of Edinburgh and the ERC ethics review procedure. |

Note that full information on the approval of the study protocol must also be provided in the manuscript.

# Flow Cytometry

## Plots

Confirm that:

- [ ] The axis labels state the marker and fluorochrome used (e.g. CD4-FITC).
- [ ] The axis scales are clearly visible. Include numbers along axes only for bottom left plot of group (a 'group' is an analysis of identical markers).
- [ ] All plots are contour plots with outliers or pseudocolor plots.
- [ ] A numerical value for number of cells or percentage (with statistics) is provided.

## Methodology

| | |
|---|---|
| Sample preparation | To estimate the size of the genome for each species we followed the protocol outlined by DeSalle et al (2005), with some minor modifications. In short, head tissue of butterflies (frozen fresh and preserved at -80 C) were ground in Gailbraith's buffer and filtered through a 40 µm mesh, resulting in a suspension of free nuclei with minimal cell debris. The solution was centrifuged at 350/500g for 1 minute, then the pellet of nuclei was resuspended in 300µl propidium idodide (50µg/ml; Sigma-Aldrich) and RNAse A (100µg/ml; Sigma-Alrich) for staining and removal of RNA. DNA content of cells were evaluated by propidium iodide binding using a 561nm excitation laser and fluorescence emission at 600-630nm. |
| Instrument | BD LSR Fortessa |
| Software | Diva v8.0.1 |
| Cell population abundance | n/a |
| Gating strategy | Each butterfly sample was measured alongside a sample of female Drosophila melanogaster (Oregon-R strain, genome size of ~175 Mb (Bennett et al 2003)) to establish a reference genome position. Single nuclei were identified by plotting area versus width for the DNA labelling with 5-50k positive nuclei recorded. For analysis, G0/1 peaks were gated for both the D. melanogaster and butterfly cells and relative intensities were then used to determine the genome size of the butterfly species using FlowJo v9.6. |

- [ ] Tick this box to confirm that a figure exemplifying the gating strategy is provided in the Supplementary Information.
