# Supplementary material for: The determinants of genetic diversity in butterflies
Source: Nat Commun. 2019 Aug 1;10:3466. doi: 10.1038/s41467-019-11308-4 (PMC6672018; doi:10.1038/s41467-019-11308-4)
Supplement: Supplementary file 4 — Description of Additional Supplementary Files [file 41467_2019_11308_MOESM4_ESM.pdf]

Description of Additional Supplementary Files

File Name: Supplementary Data 1
Description: Collection, ID and sex information for the butterfly specimens used for RNA sequencing.

File Name: Supplementary Data 2
Description: Gbif occurrence data used for estimating species ranges.

File Name: Supplementary Data 3
Description: Summaries of transcriptome assemblies and the number of 0D and 4D sites.

File Name: Supplementary Software 1
Description: Mathematica code for modeling the effect of selection on neutral diversity.
