# Supplementary material for: The determinants of genetic diversity in butterflies
Source: Nat Commun. 2019 Aug 1;10:3466. doi: 10.1038/s41467-019-11308-4 (PMC6672018; doi:10.1038/s41467-019-11308-4)
Supplement: Supplementary file 6 — Supplementary Data 2 [file 41467_2019_11308_MOESM6_ESM.pdf]

Supplementary Data 3

The following GBIF occurrence data were used to compute area ranges for 38 species of European butterfly:

References

[1] de Vries H. Observation.org, Nature data from the Netherlands. Observation.org.; 2018. https://doi.org/10.15468/5nilie.

[2] Shah M, Coulson S. Artportalen (Swedish Species Observation System). Version 92.143. ArtDatabanken; 2019. https://doi.org/10.15468/kllkyl.

[3] Blindheim T. BioFokus. Version 1.966. Natural History Museum, University of Oslo.; 2019. https://doi.org/10.15468/jxbhqx.

[4] Ranz J. Banco de Datos de la Biodiversidad de la Comunitat Valenciana. Biodiversity data bank of Generalitat Valenciana.; 2017. https://doi.org/10.15468/b4yqdy.

[5] Iturribarria M. Programa de seguimiento de mariposas diurnas del País Vasco. Version 2.5. Basque Government.; 2018. https://doi.org/10.15470/7wmd2y.

[6] Caritg R, Martínez de la Riva S. Artrópodes d'Andorra. Version 1.3. Centre d'estudis de la neu i de la muntanya d'Andorra (CENMA), Institut d'Estudis Andorrans.; 2016. https://doi.org/10.15468/d9uwhe.

[7] Beja P, Figueira R, Corley M, Grosso-Silva JM, Ferreira S, Sousa P. EDP Foz Tua: Arthropoda âĂŞ Environmental Impact Assessment [2006-2008]. Version 1.6. EDP - Energias de Portugal.; 2018. https://doi.org/10.15468/jtdrhm.

[8] Vanreusel W, Herremans M, Vantieghem P, K G, Swinnen K, Desmet P. Waarnemingen.be - Butterfly occurrences in Flanders and the Brussels Capital Region, Belgium. Version 1.6. Natuurpunt.; 2018. https://doi.org/10.15468/ezfbee.

[9] J B. The Distribution Atlas of Butterflies in Poland. Nicolaus Copernicus University of Torun; 2017. https://doi.org/10.15468/yqzyas.

[10] Uribe F. BioBlitz Barcelona 2010-14. Version 1.8. Museu de Ciéncies Naturals de Barcelona.; 2018. https://doi.org/10.15470/ssy7h3.

[11] Centre NBD. Butterflies of Ireland.; 2019. https://doi.org/10.15468/l7h1bv.

[12] Maes D, Brosens D, Beck O, Van Dyck H, Desmet P. Vlinderdatabank - Butterflies in Flanders and the Brussels Capital Region, Belgium. Version 1.4. Research Institute for Nature and Forest (INBO).; 2017. https://doi.org/10.15468/njgbmh.

[13] Telenius A, Shah M. Lepidoptera (Observations). GBIF-Sweden.; 2016. https://doi.org/10.15468/ao0ljg.

[14] Calabuig I. Atlas survey of the Butterflies of Denmark.; 2016. https://doi.org/10.15468/v5f2e2.

[15] Buchwald EC. Threatened species occurrences, Denmark 1991-2015. Version 1.4. Danish Nature Agency.; 2018. https://doi.org/10.15468/5cpovj.

[16] Moeslund JE. Vegetation data from protected areas in Denmark (§3 in the Danish Nature Protection Act). Version 8.1.Department of Bioscience, Aarhus University; 2016. https://doi.org/10.15468/ar7pbr.
